# Women’s empowerment related to pregnancy and childbirth: introduction to special issue

**DOI:** 10.1186/s12884-017-1490-6

**Published:** 2017-11-08

**Authors:** Ndola Prata, Paula Tavrow, Ushma Upadhyay

**Affiliations:** 1 University of California Global Health Institute Women’s Health, Gender, and Empowerment Center of Expertise, San Francisco, CA USA; 2 Department of Maternal and Child Health, UC Berkeley School of Public Health, 50 University Hall, Berkeley, CA USA; 3 Department of Community Health Sciences, UCLA Fielding School of Public Health, 650 Charles E. Young Drive South, Los Angeles, CA USA; 4 Department of Obstetrics, Gynecology, & Reproductive Science, University of California, San Francisco School of Medicine, San Francisco, CA USA

Empowerment is widely acknowledged as a process by which those who have been disempowered are able to increase their self-efficacy, make life-enhancing decisions, and obtain control over resources [1–3]. In addition, empowerment is multi-dimensional – a woman may be empowered in one dimension or sphere (such as financial) but not in another (such as in sexual and reproductive decision-making). Most countries now recognize the importance for girls and women to become more empowered, both as a goal in itself, as well as to achieve a more gender equitable society [4]. More recently, researchers have been assessing the contexts and mechanisms by which empowerment directly or indirectly affects various aspects of women’s health [5–7]. A better understanding of the situations where greater empowerment is associated with improved health outcomes can assist policymakers in planning and prioritizing their investments.

Although associations between women’s empowerment and some aspects of their health, such as fertility and contraception, have been studied fairly extensively and seem to be mostly positive [6, 8, 9], the relationship between women’s empowerment and pregnancy or childbirth, including abortion, has not received sufficient attention. Moreover, empowerment measures still need to be critically evaluated [10, 11] and to encompass a range of potential empowerment domains – psychological, social, political, economic and legal [8, 9, 12, 13]. The purpose of this special issue in *BMC Pregnancy and Childbirth* is to bring a multidisciplinary lens and varied methodologies to the central question of how women’s empowerment relates to pregnancy and childbirth. By highlighting women’s health concerns, rights, and empowerment, this special issue aims to catalyze societal-level changes that will yield sustainable improvements in health and well-being for women on a global scale.

This special issue is sponsored by the Women’s Health, Gender, and Empowerment Center of Expertise (COE), a part of the University of California Global Health Institute. The COE is comprised of faculty, staff and students from across the campuses of the University of California, along with practitioners and international partners. The COE promotes research, education, and community engagement at the intersection of health and empowerment in the US and globally. Collectively, it represents a wide variety of disciplines and approaches to improving women’s health and empowerment.

In the fall of 2015, the COE put out an open call for long abstracts from multiple disciplines on the role of women’s empowerment on pregnancy and childbirth. We received a total of 52 submissions, which were evaluated by all managing editors using several criteria, including strength of the empowerment construct, methodology, clarity, significance, innovation, and suitability for the supplement. The top 16 submissions were invited to submit full papers. All selected articles included a construct that is conceptualized as women’s empowerment, defined broadly. To further develop and share ideas concerning the articles for this issue, the COE conducted a one-day research workshop, which was partially funded by the National Institutes of Health, National Center for Advancing Translational Sciences, University of California, Los Angeles, Clinical and Translational Science Institute (NIH NCATS UCLA CTSI Grant Number UL1TR000124). Members of the COE submitting full papers had the opportunity to give an oral presentation presenting their study’s aims and methods, receive feedback and guidance on how to improve their study’s conceptualization, hear about other scholars’ work for this special issue, and network with others interested in these topics. A total of 12 papers successfully went through peer review and were accepted for this special issue [14].

The 12 studies included in this special issue apply methodologies from different disciplines – anthropology, sociology, law, demography, and public health – to provide empirical data on an aspect of women’s empowerment during a critical period of the reproductive life-course. The authors were also asked to discuss how their research results could affect future policies and programs. We have grouped the articles into three main subject areas, namely (1) fertility, family planning, and abortion; (2) antenatal care, delivery, and the perinatal period; and (3) maternal health and mortality.

## Empowerment and fertility, family planning, and abortion

Gipson and Upchurch [15] tried to understand intergenerational transmission of women’s empowerment by examining the influence of maternal status on the reproductive health outcomes of their daughters in the Philippines. They found that maternal empowerment was an important determinant of daughters’ timing of sexual debut, where greater empowerment led to delayed sex, regardless of whether contraception was used. However, maternal empowerment was not predictive of daughters' reports of unintended pregnancy. The authors concluded that more research is needed to better understand the intervening mechanisms between onset of sexual activity and unintended pregnancy.

While most researchers examine the impact of women’s empowerment on reproductive outcomes, Samari [16] flipped the question and innovatively investigated the impact of childbearing on women’s empowerment trajectories in Egypt. She discovered that, for a young woman, giving birth is associated with increased empowerment; the first birth and each subsequent birth predicted improvements in all measures of empowerment (individual household decision-making, joint household decision-making, and mobility), except one (financial autonomy). She also found that empowerment earlier in a woman’s life is a predictor of subsequent empowerment in life.

In her paper, McReynolds-Pérez [17] focused on Argentina, where abortion is legally restricted. Using ethnographic methods, she described the strategies used by activist healthcare providers to apply the health exception to extend the range of legal abortion. She showed how the providers conceptualized their work as opening opportunities for women to exercise their reproductive autonomy.

Mandal et al. [18] make a methodological contribution in their review of the measures of empowerment and gender-related constructs used to evaluate family planning and maternal health programs in low- and middle-income countries. Their review covered 16 program evaluations, of which only a minority used a validated measure of a gender construct. The authors recommended that future evaluations test for a clear causal pathway from program participation to an intermediary measure of gender, to the ultimate family planning or maternal health outcome that the intervention intends to improve.

## Empowerment and antenatal care, delivery, and the perinatal period

In many countries, during childbirth, women experience some form of mistreatment such as abuse, neglect, rudeness, or discrimination. Diamond-Smith et al. [19] were interested in assessing whether women in the slums of Lucknow, India, who held more gender equitable views were less likely to be mistreated. They hypothesized that empowerment could be a protective mechanism. Using the Gender Equitable Men (GEM) Scale to measure women’s views of gender equality, they found that women who had more equitable views about the role of women were less likely to report experiencing mistreatment during childbirth. Interestingly, they also discovered that the wealthiest slum women reported more mistreatment and had lower GEM scores. It is not known whether wealthier women were more likely to have higher expectations of quality, perceive slights, or experience more mistreatment. Those with higher GEM scores may be more assertive in obtaining proper treatment during childbirth.

Hoffkling et al. [20] present a rare look at the experience of transgender men in the United States who retained their uteruses, became pregnant, and gave birth. Based on in-depth interviews with 10 transgender men, the authors noted that becoming pregnant was at times an empowering act, but the experience was often difficult and alienating due to the lack of role models, transphobia and violence, insufficient training among providers, and lack of research on testosterone and pregnancy. The authors described how patient strategies and healthcare provider behaviors affected their sense of empowerment. In the end, the authors provided specific recommendations for how providers and clinics can deliver appropriate care to transgender men during the pre-transition, pre-conception, prenatal, and postpartum periods.

The objective of McGowan et al.’s [21] paper was to test the effect of the Centering Pregnancy model of group antenatal care on women’s empowerment, compared to standard individual antenatal care. The Centering Pregnancy model encompasses interactive learning and community-building, along with short individual consultations four times during a pregnancy. To assess the impact on empowerment in Malawi and Tanzania, the authors used the Pregnancy-Related Empowerment Scale, which evaluates the connectedness women feel with their caregivers, their participation in decision-making, and whether they engage in pregnancy-related healthy behaviors. They found that Centering Pregnancy seems to be empowering in Malawi, but not in neighboring Tanzania, suggesting that the model is context-dependent and may be empowering in situations where women have less access to other forms of communication, including cell phones.

Garcia and Yim [22] conducted a systematic review of studies on empowerment and interventions aimed at improving empowerment in the perinatal period. They described findings from 27 articles focusing on perinatal depressive symptoms or premature birth. All of the observational studies found significant associations between empowerment and depressive symptoms. The interventions were predominantly based on introducing the Centering Pregnancy model and most were successful in reducing preterm birth or low birthweight, but only interventions that provided women with coping skills for future stressors reduced women’s perinatal depressive symptoms.

In their literature review, Afulani et al. [23] examined the links between women’s empowerment and prematurity. Although they did not find evidence supporting a direct link between women’s empowerment and prematurity, they did identify some studies that linked empowerment to factors known to be associated with prematurity and outcomes for premature babies, namely (1) preventing early marriage and promoting family planning, which will delay first pregnancy and increase inter-pregnancy intervals; (2) improving women’s nutritional status; (3) reducing domestic violence and other factors associated with stress; and (4) promoting use of recommended health services during pregnancy and delivery to help prevent prematurity and improve survival of their babies. Thus, improving women’s empowerment could potentially prevent prematurity, but definitive proof is still lacking.

## Empowerment and maternal health and mortality

In their article, Shimamoto and Gipson [24] examined the mechanisms by which women’s status and empowerment affect skilled birth attendant use in West Africa. They found the structural equation modeling approach to be useful in examining the complex and multidimensional constructs of women’s empowerment and their effects. Despite variations across measures, many of the women’s status and empowerment variables were positively associated with skilled birth attendance. In particular, women’s education demonstrated a substantial indirect effect, and higher education was related to older age at first marriage, which in turn was associated with higher levels of empowerment and the use of skilled birth attendants. Interestingly, the authors did not find significant associations between household decision-making and the use of skilled birth attendance.

It is commonly believed that greater women’s empowerment will lead to improvements in their health, particularly in areas where disparities are highest such as maternal mortality. To test this assumption, Lan and Tavrow [25] sought to assess various gender composite measures to determine if they were associated with reduced mortality at the national level, after controlling for other macro-level and direct determinants. They used data from 44 low-income countries, half of which are in Africa. After controlling for all measures, they found that none of the composite measures of gender equality were significantly linked to maternal mortality in these countries. Rather, skilled birth attendance was the main factor associated with maternal mortality in non-African countries, and perceptions of corruption were most linked to mortality in African countries, where mortality is highest. They concluded that improving gender equality and even skilled birth attendance is unlikely to reduce maternal mortality in Africa unless corruption is addressed.

Laws and social norms can interact to disempower women, or they can be used to empower them. In addition, laws often have a norm-setting function. In their paper, Dunn et al. [26] analyzed the impact of international and domestic decisions on access to high quality reproductive healthcare, showing that human rights litigation can support other efforts to achieve better care for women. They discussed several case studies in which national courts in countries such as Uganda, as well as international treaty bodies, have challenged traditional structures that discriminate against women. They argued that human rights litigation is a women’s empowerment strategy that needs greater attention, because they found that cases like Alyne v. Brazil brought public awareness about discrimination against poor or marginalized women in the health system and provided leverage to civil society to make changes. Indeed, human rights litigation often complements political and social movements and provides momentum to bring change.

Through an overview of the collection of articles as a whole, the key findings were:

1. Fertility, pregnancy and abortion
  Fertility decline does seem to be linked to better well-being for women, but patriarchal gender norms can inhibit its impact. Just as empowerment seems to affect health, women who start childbearing later are more likely to show more gender equitable attitudes. When mothers are empowered, their daughters are less likely to have sex at a young age, but they still have the same rates of unintended pregnancies. Among slum women, higher rates of expressed empowerment are correlated with lower levels of mistreatment by health providers during delivery. Providers who are themselves empowered can actively expand women’s access to abortion, even in countries where it is legally restricted. Overall, gender-integrated interventions related to family planning and maternal health are not evaluated with sufficiently consistent and validated measures of women’s empowerment to know if they are having the intended impact.
2. Antenatal care, delivery, and the perinatal period
  In some contexts, group antenatal care can be more empowering to women than the standard of care, possibly because it increases communication and learning among a peer group. Pregnant women who feel empowered through better coping skills prior to birth seem less likely to suffer from postpartum depression. For transgender men who give birth, culturally competent and caring providers can help to make the experience more empowering, although transphobia in society can make these men feel alienated and anxious. While a direct link cannot be found between disempowerment and low birthweight or premature births, the same programs that empower women (such as programs to reduce intimate partner violence) can also be expected to reduce prematurity.
3. Maternal health and mortality
  Women who are more empowered are more likely to use skilled birth attendants, which could be expected to lower maternal mortality. However, in Africa, women’s empowerment may not lead to changes in maternal mortality rates if health systems remain corrupt. Litigation can be an empowering strategy globally if it reframes maternal mortality as discriminatory and changes public norms.

In summary, this special issue provides a platform for examining the relevance of empowerment to various features of women’s (and transgender men’s) experiences of pregnancy and childbirth across the globe. While women’s empowerment itself still needs further conceptualization, this special issue broadens the range of health outcomes that are often associated with empowerment, provides insights into the current state of knowledge and research, and points to the importance of considering and measuring empowerment when designing and implementing programs.

We express our deepest gratitude to Chiao-Wen Lan for managing all steps of the editorial process and ensuring that the authors received constructive, impartial reviews. We are also grateful for the time and invaluable comments provided by the peer reviewers of this special issue (those reviewers with an asterisk are also members of the COE):

- Onyema Afulukwe, Center for Reproductive Rights
- Koki Agarwal, Jhpiego – an affiliate of Johns Hopkins University
- Saifuddin Ahmed, Johns Hopkins University
- Meg Autry,* University of California, San Francisco
- Sarah Baum, Ibis Reproductive Health
- Joelle Brown,* University of California, San Francisco
- Julianna Deardorff, University of California, Berkeley
- Teresa DePineres, Fundación Oriéntame/ESAR
- Shari Dworkin,* University of California, San Francisco
- Linda Franck,* University of California, San Francisco
- Caitlin Gerdts, Ibis Reproductive Health
- Sarah Jane Holcombe,* University of California, Berkeley
- Rana Marie Jaleel,* University of California, Davis
- Randall Kuhn, University of California, Los Angeles
- Andrzej Kulczycki, University of Alabama, Birmingham
- Susan Meffert,* University of California, San Francisco
- Deborah Mindry,* University of California, Los Angeles
- Corrina Moucheraud,* University of California, Los Angeles
- Kavita Singh Ongechi, University of North Carolina at Chapel Hill
- Bhavya Reddy, Public Health Foundation of India
- Lara Stemple,* University of California, Los Angeles
- Kirsten Stoebenau, American University
- Dallas Swendeman,* University of California, Los Angeles
- Charlotte Warren, Population Council
- Sheri Weiser,* University of California, San Francisco
- Mellissa Withers,* University of Southern California

## Additional file

Additional file 1: Open peer review. (PDF 155 kb)
