# Supplementary material for: Women’s empowerment related to pregnancy and childbirth: introduction to special issue
Source: BMC Pregnancy Childbirth. 2017 Nov 8;17(Suppl 2):352. doi: 10.1186/s12884-017-1490-6 (PMC5688486; doi:10.1186/s12884-017-1490-6)
Supplement: Additional file 1: — Open peer review. (PDF 155 kb) [file 12884_2017_1490_MOESM1_ESM.pdf]

Reviewer report

Title: Women's empowerment related to pregnancy and childbirth: introduction to special issue

Reviewer: Hora Soltani

Declaration of competing interests: I have no conflicting interest in reviewing this manuscript.

Women's empowerment related to pregnancy and childbirth: introduction to special issue

by
Ndola Prata, Paula Tavrow and Ushma Upadhyay

Empowerment is widely acknowledged as a process by which those who have been disempowered are able to increase their self-efficacy, make life-enhancing decisions and obtain control over resources [1-3]. In addition, empowerment is multi-dimensional: a woman may be empowered in one dimension or sphere (such as financial) but not in another (such as in in sexual and reproductive decision-making). Most countries now recognize the importance for girls and women to become more empowered--both as an endpoint in itself, as well as to achieve a more gender equitable society [4]. More recently, researchers have been assessing the contexts and mechanisms by which empowerment directly or indirectly affects various aspects of women's health [5-7]. Understanding better the situations where greater empowerment is associated with improved health outcomes can assist policy-makers in planning and prioritizing their investments.

~~Whereas~~ Although associations between women's empowerment and some aspects of their health, such as fertility and contraception, have been studied fairly extensively and seem to be mostly positive [6, 8, 9], the relationship between women's empowerment and pregnancy or childbirth, including abortion, has not received sufficient attention. Moreover, empowerment measures used still need to be critically evaluated [10, 11] and to include the range of influences on women's health, including the psychological, political and legal [8, 9, 12, 13]. The purpose of this special issue to the *BMC Pregnancy and Childbirth* is to bring a multi-disciplinary lens and varied methodologies to the central question of how **women's empowerment relates to pregnancy and childbirth.** By highlighting women's health concerns, rights, and empowerment, this special issue aims to catalyze societal-level changes that will yield sustainable improvements in health and well-being for women on a global scale.

This special issue is sponsored by the Center of Expertise on Women's Health, Gender and Empowerment (COE), a part of the University of California Global Health Institute. The COE is comprised of faculty, staff and students from ~~the~~ across the campuses of the University of California, along with practitioners and international partners. The COE promotes research, education, and community engagement at the intersection of health and empowerment in the US and globally. Collectively, it represents a wide variety of disciplines and approaches to improving women's health and empowerment.

In the fall of 2015 the COE put out an open call for papers from multiple disciplines that focus on the role of women's empowerment in pregnancy and childbirth. We received a total of 52 submissions of which 13 successfully went through peer review and were accepted for this special issue [14]. All selected articles included a construct that is conceptualized as women's empowerment, defined broadly. To further develop and share ideas concerning the articles for this issue, the COE conducted a one-day research workshop, which was partially funded by NIH NCATS UCLA CTSI (Grant Number UL1TR000124). Members of the COE submitting full papers had the opportunity to give an oral presentation presenting their study's aims and methods, receive feedback and guidance on how to improve their study's conceptualization, hear about other scholars' work for this special issue, and network with others interested in these topics.

Comment [HS1]: As this is an international journal, local/national phrases should be avoided. Perhaps "Autumn" instead of "fall", would be more suitable and understandable for a wider audience

Comment [HS2]: A large number of articles were rejected before peer review- was there a clear criteria for that? Who decided and on what basis? Were they judged based on relevance or quality-I suppose it is important for the readers and particularly those who submitted to know this

Comment [HS3]: Doesn't this need to be in full when appeared first in the text?

The 13 studies included in this special issue apply methodologies from different disciplines—anthropology, sociology, law, demography and public health—to provide empirical data on an aspect of women's empowerment during a critical period of the reproductive life-course. The articles in this collection focus primarily on countries and contexts where women's empowerment is considered highly relevant to reproductive health. The authors were also asked to discuss how their research results could affect future policies and programs. We have grouped the articles into three main subject areas that span from pre-conception to delivery, including mortality: (1) fertility, family planning and abortion; (2) antenatal care, delivery and the perinatal period; and (3) maternal health and mortality.

Comment [HS4]: I am not sure this is quite clear-it would be helpful to give examples

Comment [HS5]: Does this mean the categorization is based on intervention delivery, implementation period or outcome measurements-the sentence needs to be clarified

1. Empowerment & fertility, family planning and abortion

In their examination of fertility decline influence on gender equality in Tamil Nadu, India, **Pande and colleagues** analyze surveys and censuses over multiple decades. They find that empowerment and gender equality in marriage norms and dowry patterns remain ambivalent. However, the decline in fertility does seem to have brought about more gender equality in education and employment, as well as enhanced well-being for women. Increased time away from childbearing and a change in the value of children may be the pathways through which improvements in women's welfare occurred. The authors argue that the full impact of fertility decline on women's lives in Tamil Nadu will only be felt when traditional gender norms concerning women's status also change significantly.

Gipson & Upchurch seek to understand intergenerational transmission of women's empowerment. They do so by examining the influence of mother's status on the reproductive health outcomes of their daughters in the Philippines. They find that mother's empowerment is an important determinant of daughter's timing of sexual debut: greater empowerment delayed sex, regardless of whether contraception was used. However, mother's empowerment was not predictive of daughter's reports of an unintended pregnancy. The authors conclude that more research is needed to better understand the intervening mechanisms between onset of sexual activity and unintended pregnancy.

While most researchers examine the impact of women's empowerment on reproductive outcomes, **Samari** flips the question and innovatively investigates the impact of childbearing on women's empowerment trajectories in Egypt. She discovers that for a young woman, giving birth is associated with increased empowerment; the first birth and each subsequent birth predicted improvements in all measures of empowerment except financial autonomy. She also finds that women's earlier empowerment is a predictor of subsequent empowerment in life.

Comment [HS6]: What are these other elements of empowerment

Comment [HS7]: This is not very clear, is this indicating sustainability? What does early or late empowerment mean? an indication of relative timing/period makes this statement clearer

In her paper, **McReynolds-Pérez** focuses on Argentina where abortion is legally restricted. Using ethnographic methods, she describes the strategies used by activist health care providers to apply the health exception to extend the range of legal abortion. She shows how the providers conceptualized their work as opening opportunities for women to exercise their reproductive autonomy.

Mandal and colleagues make a methodological contribution in their review of the measures of empowerment and gender-related constructs used to evaluate family planning and maternal health programs in low and middle income countries. Their review covers 16 program evaluations, of which only a minority used a validated measure of a gender construct. The authors recommend that future evaluations test for a clear causal pathway from program participation to an intermediary measure of gender, to the ultimate family planning or maternal health outcome that the intervention intends to improve.

2. Empowerment & antenatal care, delivery and the perinatal period

In many countries, during childbirth women experience some form of mistreatment--such as abuse, neglect, rudeness, or discrimination. **Diamond-Smith and colleagues** were interested in assessing whether women in the slums of Lucknow, India, who held more gender equitable views were less likely to be mistreated. They hypothesize that empowerment could be a protective mechanism. Using the Gender Equitable Men (GEM) Scale to measure women's views of gender equality, they find that women who had more equitable views about the role of women were less likely to report experiencing mistreatment during childbirth. Interestingly, they also discover that the wealthiest slum women reported more mistreatment and had lower GEM scores. This suggests that wealthier women are more likely to perceive slights or experience more mistreatment. Those with higher GEM scores may be more assertive in obtaining proper treatment during childbirth.

Comment [HS8]: Could this be related to their level of expectation rather than lower GEM-how the confounding factors such as expected norm is adjusted for?

Hoffkling and colleagues present a rare look at the experience of transgender men in the United States who retained their uteruses, became pregnant, and gave birth. Based on in-depth interviews with 10 transgender men, the authors noted that becoming pregnant was at times an empowering act, but the experience was often difficult and alienating due to the lack of role models, transphobia and violence, insufficient training among providers, and lack of research on testosterone and pregnancy. The authors describe how patients' strategies, and health care providers' behaviors, affected their sense of empowerment. In the end, the authors provide specific recommendations for how providers and clinics can provide appropriate care to transgender men during the pre-transition, pre-conception, prenatal, and postpartum periods.

The objective of **McGowan and colleagues'** paper is to test the effect of the Centering Pregnancy model of group antenatal care on women's empowerment, compared to standard individual antenatal care. The Centering Pregnancy model encompasses interactive learning and community-building, along with short individual consultations four times during a pregnancy. To assess the impact on empowerment in Malawi and Tanzania, the authors use the Pregnancy-Related Empowerment Scale (PRES), which evaluates the connectedness women feel with their caregivers, their participation in decision-making, and whether they engage in pregnancy-related healthy behaviors. They find that Centering Pregnancy seems to be empowering in Malawi, but not in neighboring Tanzania. This suggests that the model is context-dependent and may be empowering in situations where women have less access to other forms of communication, including cell phones.

Garcia & Yim conduct a systematic review of studies on empowerment and interventions aimed at improving empowerment in the perinatal period. They describe findings from 27 articles focusing on perinatal depressive symptoms or premature birth. All of the observational studies find significant associations between empowerment and depressive symptoms. The interventions were predominantly based on introducing the Centering Pregnancy model. Most were successful in reducing preterm birth or low birthweight, but only interventions that provided women with coping skills for future stressors reduced women's perinatal depressive symptoms.

In their literature review, **Afulani and colleagues** examine the links between women's empowerment and prematurity. Although they do not find evidence supporting a direct link between women's empowerment and prematurity, they do identify some studies that linked empowerment to factors known to be associated with prematurity and outcomes for premature babies, namely: 1) preventing early marriage and promoting family planning which will delay first pregnancy and increase inter-pregnancy intervals; 2) improving women's nutritional status; 3) reducing domestic violence and other factors associated with stress; and 4) promoting use of recommended health services during pregnancy and delivery to help prevent prematurity and improve survival of their babies. Thus, improving women's empowerment could potentially prevent prematurity, but definitive proof is still lacking.

Comment [HS9]: What about the nature/content or the characteristics of providers -were they identical or the differences may contribute to observed differences in empowerment?

Comment [HS10]: These are very important and interesting, reminding me of a recently updated Cochrane review focused on evaluating Midwife-led continuity model of care with other models of care showing a reduction in premature birth! The philosophy of Midwife-led care is based on advocacy, building relationship and promoting normality and the relationship midwife builds with the women on the basis of empowering them and enhancing their feeling of being in control [Sandall J, **Soltani H**, Gates S, Shennan A, Devane D. (2016) Midwife-led continuity models versus other models of care for childbearing women. Cochrane Database of Systematic Reviews 2016, Issue 4. Art. No.: CD004667. DOI: 10.1002/14651858.CD004667.pub5] The results of this review, highlights the importance of the professionals and organisation of maternity care and its empowering aspects having health benefits for mothers and their babies (significant reduction in preterm birth-which is in line with these statements. Just a side observation for the information of authors in confirmation of their statements highlighting the importance of maternity care organisation and its impact on empowerment and improved birth outcomes

3. Empowerment & maternal health and mortality

In their article, **Shimamoto & Gipson** examine the mechanisms by which women's status and empowerment affect skilled birth attendant use in West Africa. They find the structural equation modeling approach to be useful in examining the complex and multidimensional constructs of women's empowerment and their effects. Despite noting variations across measures, many of the women's status and empowerment variables are positively associated with skilled birth attendance. In particular, women's education demonstrates a substantial indirect effect and higher education is related to older age at first marriage, which is in turn associated with higher levels of empowerment and the use of skilled birth attendants. Interestingly, authors do not find significant associations between household decision-making and the use of skilled birth attendance.

It is commonly believed that greater women's empowerment will lead to improvements in their health, particularly in areas where disparities are highest such as maternal mortality. To test this assumption, **Lan & Tavrow** sought to assess various gender composite measures to determine if they were associated with reduced mortality at the national level, after controlling for other macro-level and direct determinants. They use data from 44 low income countries, half of which were in Africa. After controlling for all measures, they find that none of the composite measures of gender equality were significantly linked to maternal mortality in these countries. Rather, skilled birth attendance was the main factor associated with maternal mortality in non-African countries, and perceptions of corruption were most linked to mortality in African countries, where mortality is highest. They conclude that improving gender equality and even skilled birth attendance is unlikely to reduce maternal mortality in Africa unless corruption is addressed.

Laws and social norms can interact to disempower women, or they can be used to empower women. In addition, laws often have a norm-setting function. In their paper, **Dunn and colleagues** analyze the impact of international and domestic decisions on access to high quality reproductive health care, showing that human rights litigation can support other efforts to achieve better care for women. They discuss several case studies in which national courts in countries such as Uganda, as well as international treaty bodies, have challenged traditional structures that discriminate against women. They argue that human rights litigation is a women's empowerment strategy that needs more attention, because they find that cases like *Alyne v. Brazil* bring public awareness about the discrimination against poor or marginalized women in the health system, and provide leverage to civil society to make changes. Indeed, human rights litigation often complements political and social movements and provides momentum to bring change.

Looking at the collection of articles as a whole, the key findings seem to be:

1. Fertility, pregnancy and abortion
    - Fertility decline does seem to be linked to better well-being for women, but patriarchal gender norms can inhibit its impact. Just as empowerment seems

to affect health, women who start childbearing later are more likely to show more gender equitable attitudes. When mothers are empowered, their daughters are less likely to have sex at a young age but they still have the same rates of unintended pregnancies. Among slum women, higher rates of expressed empowerment are correlated with lower less of mistreatment by health providers during delivery. Providers who are themselves empowered can actively expand women's access to abortion, even in countries where it is legally restricted. Overall, gender-integrated interventions related to family planning and maternal health are not evaluated with sufficiently consistent and validated measures of women's empowerment to know if they are having the intended impact.

2. Antenatal care, delivery, and the perinatal period
   - In some contexts, group antenatal care can be more empowering to women than the standard of care, possibly because it increases communication and learning among a peer group. Pregnant women who feel empowered through better coping skills prior to birth seem less likely to suffer from post-partum depression. For transgender men who give birth, culturally competent and caring providers can help to make the experience more empowering, although transphobia in society can make these men feel alienated and anxious. While a direct link cannot be found between disempowerment and low birthweight or premature births, the same programs that empower women (such as programs to reduce intimate partner violence) also can be expected to reduce prematurity.

3. Maternal health and mortality
   - Women who are more empowered are more likely to use skilled birth attendants, which could be expected to lower maternal mortality. However, in Africa, women's empowerment may not lead to changes in maternal mortality rates if health systems remain corrupt. Litigation can be an empowering strategy globally if it reframes maternal mortality as discriminatory and changes public norms.

In summary, this special issue provides a platform for examining the relevance of empowerment to various features of women's (and transgender men's) experiences of pregnancy and childbirth across the globe. While women's empowerment itself still needs further conceptualization, this special issue broadens the range of health outcomes that are

Comment [HS11]: is this lower mistreatment or lower less of mistreatment? Is this consistent with the statement on the top of page 5 about wealthiest slum women-please make it simper for the readers to follow

Comment [HS12]: Was there any indication of models of maternity care and principles of continuity of care/building respectful relationship with the mothers which could have mediated the empowering aspects to enhance birth outcomes?

often associated with empowerment, provides insights into the current state of knowledge and research, and points to the importance of considering and measuring empowerment when designing and implementing programs.

We express our deepest gratitude to Chiao-Wen Lan for managing all steps of the editorial process and ensuring that the authors received constructive, impartial reviews. We are also grateful for the time and invaluable comments provided by the peer reviewers of this special issue (Those reviewers with an asterisk are also members of the COE):

Onyema Afulukwe, Center for Reproductive Rights
Koki Agarwal, Jhpiego – an affiliate of Johns Hopkins University
Saifuddin Ahmed, Johns Hopkins University
Meg Autry,* University of California, San Francisco
Sarah Baum, Ibis Reproductive Health
Joelle Brown,* University of California, San Francisco
Julianna Deardorff, University of California, Berkeley
Teresa DePineres, Fundación Oriéntame/ESAR
Shari Dworkin,* University of California, San Francisco
Linda Franck,* University of California, San Francisco
Caitlin Gerdts, Ibis Reproductive Health
Sarah Jane Holcombe,* University of California, Berkeley
Rana Marie Jaleel,* University of California, Davis
Randall Kuhn, University of California, Los Angeles
Andrzej Kulczycki, University of Alabama, Birmingham
Susan Meffert,* University of California, San Francisco
Deborah Mindry,* University of California, Los Angeles
Corrina Moucheraud,* University of California, Los Angeles
Kavita Singh Ongechi, University of North Carolina at Chapel Hill
Bhavya Reddy, Public Health Foundation of India
Lara Stemple,* University of California, Los Angeles
Kirsten Stoebenau, American University
Dallas Swendeman,* University of California, Los Angeles
Charlotte Warren, Population Council
Sheri Weiser,* University of California, San Francisco
Mellissa Withers,* University of Southern California

References:

1. Kabeer N: **Reflections on the measurement of women's empowerment**. In: *Discussing Women's Empowerment—Theory and Practice.* edn. Stockholm: Novum Grafiska AB; 2001.
2. Malhotra A, Schuler SR, Boender C: **Measuring Women's Empowerment as a Variable in International Development**. In. Washington, DC; 2002: 58.
3. Sen G: **Womens empowerment and human rights: the challenge to policy**. 1994.
4. Klugman J, Hanmer L, Twigg S, Hasan T, McCleary-Sills J, Santamaria J: **Voice and agency: Empowering women and girls for shared prosperity**: World Bank Publications; 2014.
5. Pratley P: **Associations between quantitative measures of women's empowerment and access to care and health status for mothers and their children: A systematic review of evidence from the developing world**. *Soc Sci Med* 2016, **169**:119-131.
6. James-Hawkins L, Peters C, VanderEnde K, Bardin L, Yount KM: **Women's agency and its relationship to current contraceptive use in lower- and middle-income countries: A systematic review of the literature**. *Glob Public Health* 2016:1-16.
7. Ahmed S, Creanga AA, Gillespie DG, Tsui AO: **Economic Status, Education and Empowerment: Implications for Maternal Health Service Utilization in Developing Countries**. *PLoS One* 2010, **5**(6):e11190.
8. Upadhyay UD, Gipson JD, Withers M, Lewis S, Ciaraldi EJ, Fraser A, Huchko MJ, Prata N: **Women's empowerment and fertility: a review of the literature**. *Soc Sci Med* 2014, **115**:111-120.
9. Prata N, Fraser A, Huchko MJ, Gipson JD, Withers M, Lewis S, Ciaraldi EJ, Upadhyay UD: **Women's Empowerment and Family Planning: A Review of the Literature**. *J Biosoc Sci* 2017:1-31.
10. Upadhyay UD, Karasek D: **Women's Empowerment and Achievement of Desired Fertility in Sub-Saharan Africa. DHS Working Papers No. 80**. In. Calverton, Maryland: ICF Macro; 2010.
11. Heckert J, Fabic MS: **Improving Data Concerning Women's Empowerment in Sub-Saharan Africa**. *Studies in family planning* 2013, **44**(3):319-344.
12. Shimamoto K, Gipson JD: **The relationship of women's status and empowerment with skilled birth attendant use in Senegal and Tanzania**. *BMC Pregnancy Childbirth* 2015, **15**:154.
13. Mitroi LR, Sahak M, Sherzai AZ, Sherzai D: **The Women's Health Care Empowerment Model as a Catalyst for Change in Developing Countries**. *Health Care Women Int* 2016, **37**(3):273-287.
14. Center of Expertise on Women's Health & Empowerment: **Call for papers: BMC Pregnancy and Childbirth**. http://www.ucghi.universityofcalifornia.edu/news-events/items/2015-call-for-papers-bmc-pregnancy.aspx. Accessed on January 31, 2017.
